# Microbiological Profile and Outcome of Surgical Site Infections Following Orthopedic Surgeries in a Tertiary Care Hospital

**DOI:** 10.7759/cureus.76874

**Published:** 2025-01-03

**Authors:** Adrita Das, Sumanyu K Tripathy, Ipsa Mohapatra, Nirmala Poddar, Dipti Pattnaik, Sayashi S, Kumudini Panigrahi

**Affiliations:** 1 Department of Microbiology, Kalinga Institute of Medical Sciences, Bhubaneswar, IND; 2 Department of Orthopedics, Kalinga Institute of Medical Sciences, Bhubaneswar, IND; 3 Department of Community Medicine, Kalinga Institute of Medical Sciences, Bhubaneswar, IND

**Keywords:** healthcare-associated infection (hai), hospital infection control, infection, orthopaedic surgery, surgical site infection(ssi)

## Abstract

Surgical site infections (SSIs) are one of the most common adverse events that occur in hospitalized patients undergoing surgical procedures or in outpatient surgical measures, regardless of the advances in preventive procedures. SSI may lead to disastrous consequences in orthopedic practice as it may involve the joints and bones and is extremely difficult to get rid of the infection. The present study was designed to evaluate the rates, risk factors, microbiological profiles, and outcomes of SSIs following orthopedic procedures in patients admitted to a tertiary care hospital in Eastern India during the study period of September 2022 to March 2024. A total of 1327 patients who underwent orthopedic surgeries were followed up for the development of SSI, among whom 105 (7.9%) developed SSI, making an incidence rate of 7.9%. The incidence of SSI in different surgeries was 9.5% (34/359) in closed reduction with fixation, 8.5% (65/766) in open reduction with internal fixation, 4.4% (3/69) in hip arthroplasty, and 2.3% (3/133) in knee arthroplasty. Maximum (27.6%) patients having SSI were of the age group of 20-29 years, and 87.62% were males. The habit of smoking was found to be highly statistically significant. The common gram-positive organisms isolated were *Staphylococcus aureus* and *Enterococcus* species, which were mostly sensitive to vancomycin, linezolid, teicoplanin, and tigecycline. The common gram-negative organisms isolated were *Klebsiella pneumoniae, Pseudomonas* species*, Escherichia coli, and Acinetobacter species,* many of which were multidrug-resistant organisms and were sensitive to amikacin, amoxicillin-clavulanate, and ceftriaxone.

## Introduction

Nosocomial infections (NIs) are a concern of public health importance that have a considerable impact both at the individual level and at the economic level. Nosocomial, also known as hospital-acquired, infections occur during hospital stays and are not present during hospital admission. Surgical site infections (SSIs) are the leading cause of NI among operated patients. Improving the surveillance and prevention of SSIs is part of the national program to fight NI [1].

SSIs are one of the most common healthcare-associated infections (HCAIs), causing substantial morbidity, mortality, increased cost of treatment, and severe psychosocial consequences to the patients and families.

The figures of SSI burden are likely to be highly underestimated because most hospitals truncate SSI surveillance after the discharge of the patient from the hospital, as many infections emerge following discharge. So robust post-discharge surveillance is required using trained and committed personnel to generate accurate data [1-5].

In India, there is no existing system for systematic surveillance of SSIs, encompassing the post-discharge period. To fill this knowledge gap, we proposed to estimate the proportion of SSIs occurring during admission and after discharge in the context of an orthopedic trauma setting. This study aimed to evaluate the rates, risk factors, microbiological profiles, and outcomes of SSIs following orthopedic procedures in patients admitted to a tertiary care hospital in Eastern India.

## Materials and methods

Study setting and study participants

A prospective cohort study was conducted from September 2022 to March 2024 at the Department of Microbiology in collaboration with the Department of Orthopedics of Kalinga Institute of Medical Sciences, Bhubaneswar, India, after getting approval from the Institutional Ethics Committee (Ref no.: KIIT/KIMS/IEC/951/2022). Written informed consent was obtained from all participants, ensuring confidentiality. The research included all patients undergoing orthopedic surgeries during the specified period, excluding those with osteomyelitis, infected implants, immunodeficiency, or prior surgeries at other hospitals.

Sample size and sampling technique

**Figure 1:**
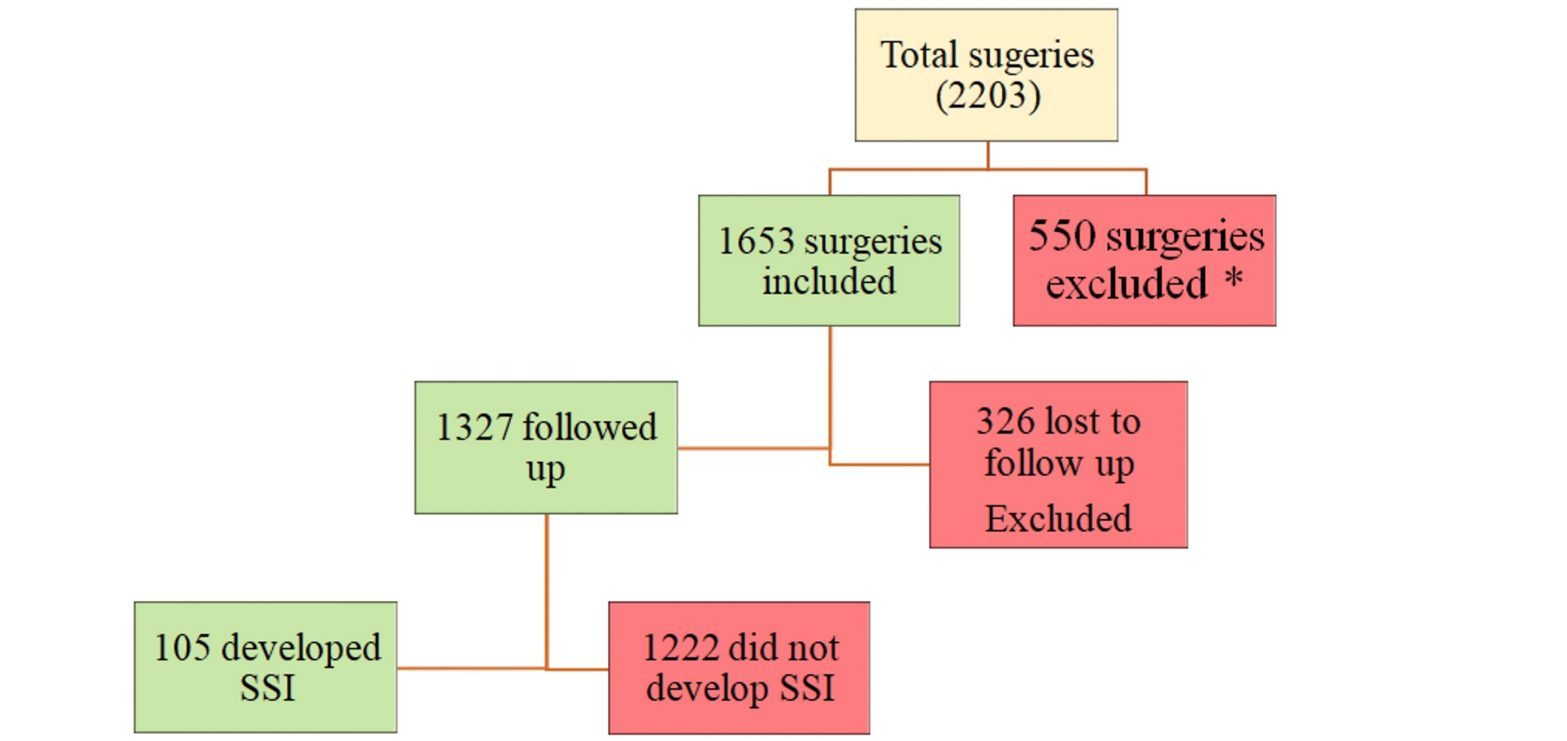
Sampling method Surgeries performed for osteomyelitis, infected implant/ prosthesis, septic bone, and joint surgeries, immunodeficiency, prior surgeries at other facilities were excluded from the study (550 samples)

From September 1, 2022, to March 31, 2024, a total of 2,203 patients underwent surgery. Of these, 1,653 patients met the inclusion criteria for the study. Out of the 1,653 patients, 1,327 were successfully followed up. Among them, 105 were diagnosed with surgical site infections (SSIs), resulting in a final sample size of 105 (Figure 1). Due to the feasibility of conducting follow-up of patients in the single tertiary care hospital, a convenience sampling method was used for the study. (Figure 1)

Data collection

Data was collected using a structured form for each surgery, capturing patient demographics, comorbidities, site of fracture, type of surgery, and various preoperative and intraoperative factors. Specific preoperative factors included hospital stay duration, blood glucose levels, bathing practices, antimicrobial prophylaxis, hair removal, skin preparation, American Society of Anesthesiologists (ASA) score, and surgical hand preparation. Intraoperative factors encompassed the nature of the operation, wound class, suture material used, duration of operation, and the number of personnel in the operating theater. For patients who developed SSIs, a numerator form documented microbiological data, including the timing of SSI detection, culture and sensitivity reports, and clinical outcomes.

Methodology

Post-surgery, surgical wounds were inspected for signs of infection on the third and fifth days and at discharge. Patients received post-discharge care instructions. Follow-up data were collected via telephone interviews at 30, 60, and 90 days post-operation, asking about symptoms such as fever, discharge characteristics, swelling, delayed healing, and any new antibiotic taken. SSIs were diagnosed according to CDC criteria, with endpoints including superficial, deep, organ/space infections, or no SSI.

Wounds were classified based on CDC criteria: closed fractures as clean and open fractures as clean-contaminated, contaminated, or dirty based on preoperative and intraoperative findings. Types of surgeries included open reduction and internal fixation (ORIF), closed reduction with internal fixation (CRIF), and arthroplasties (hip and knee). If SSIs were suspected, pus, aspirate, or wound swabs were collected aseptically and transported to the microbiology lab at 2-8°C for processing [6].

Sample collection and processing

Sample was either pus, aspirate, or wound swab from surgical site infection. Microbiological processing of samples was done for gram staining and culture sensitivity testing. Samples for culture were inoculated onto 5% sheep blood agar and MacConkey agar and incubated aerobically at 37°C for 18-24 hours. If no growth occurred, subcultures were performed. Isolated colonies were identified, and an antimicrobial susceptibility test was conducted by the VITEK 2 automated system. Quality control strains were utilized for accuracy.

Multi-drug-resistant organisms were defined as those resistant to at least one agent in three or more antimicrobial categories, while carbapenem-resistant *Enterobacterales* exhibited resistance to at least one carbapenem.

Data analysis and statistical analysis

The outcomes of SSIs were evaluated based on hospital stay duration, readmissions, mortality, and costs attributed to SSI. Statistical analysis was performed using EpiInfo software (version 7.2.3.1, Centers for Disease Control and Prevention (CDC), Atlanta, Georgia, US), with categorical data described using frequency distributions and percentages. Continuous data were summarized using mean, standard deviations, median, and interquartile range. In bivariate analysis, the outcome variable (SSI) and explanatory variables were expressed as odds ratios (OR) with 95% confidence intervals (CI); all variables having a p-value ≤ 0.05 in the bivariate analysis were taken as significant associations between the explanatory and outcome variables, applying the appropriate tests of association (t-test, Fischer’s exact test, and chi-square test).

## Results

Among 105 participants who developed SSI, their mean age was 37.13 ± 15.88 years with a range of nine to 76 years; the median age was 36 years with an interquartile range (IQR) of 25 years to 50 years. Around 87.62% (92/105) were males, and 27.6% (29/105) were of the age group of 20-29 years (Table 1).

**Table 1:** Demographic profile of patients with SSI (n=105)

Demographic variable	Patients with SSI	
	Frequency in number	Frequency in %
Age group (in years)		
0-9	2	1.9
10-19	12	11.4
20-29	29	27.6
30-39	17	16.1
40-49	18	17.1
50-59	18	17.1
60-69	7	6.6
70-80	2	1.9
Gender		
Male	92	87.62
Female	13	12.38

**Figure 2:**
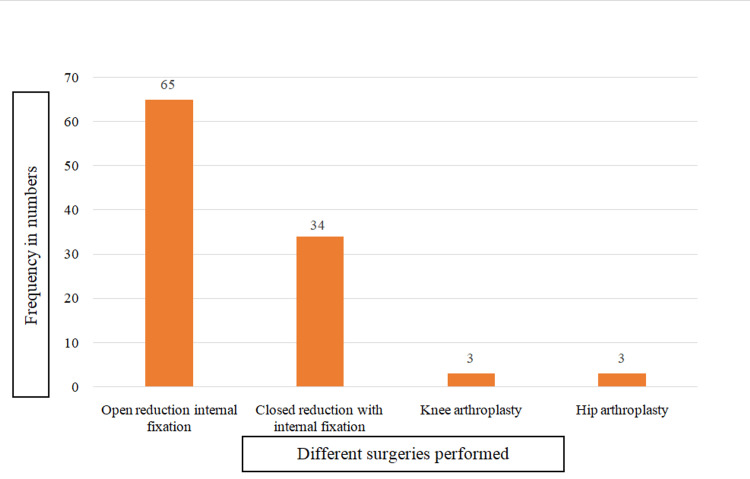
Number of SSI in different surgeries (n=105) SSI: surgical site infections

During the study period, the total number of surgeries followed up for the development of SSI was 1327, among whom 105 (7.9%) developed SSI, making an incidence rate of 7.9%. Among the surgeries performed, open reduction with internal fixation (ORIF), knee arthroplasty, hip arthroplasty, and closed reduction with internal fixation (CRIF) were 766 (57.72%), 133 (10.02%), 69 (5.1%), and 359 (27.0%), respectively. Although the maximum number of surgical procedures were ORIF, the SSI incidence rate was found to be highest, i.e., 9.5% (34/359), in CRIF, followed by 8.5% (65/766) in ORIF, 4.4% (3/69) in hip arthroplasty, and 2.3% (3/133) in knee arthroplasty. Figure 2 shows the numbers of SSI following different orthopedic surgeries.

Table 2 shows the non-modifiable patient factors, age and gender, in both groups. Considering the age groups, 9.74% of those <21 years reported a higher number of SSIs, whereas those >60 years reported around 3.43%; the elderly (>60 years) had a lower incidence of SSI, and this difference was also found to be statistically significant with a p-value of 0.005. Among females, 3.39% developed SSI, whereas among males, 9.75% had SSI. This difference was also found to be statistically significant with a p-value of 0.0001.

**Table 2:** Non-modifiable patient factors associated with SSI the column total percentages are shown in brackets; *applying chi-square test, degree of freedom=1 SSI: surgical site infection; CI: confidence interval

Variables	Total no. of patients (1327)	Patients with SSI (105)	Patients with no SSI (1222)	Odds ratio (95% CI)	Tests of association	P-value
Age group (in years)						
<21	154	15 (9.74%)	139 (90.26%)	1.29 (0.73-2.31)	0.54*	0.37
21-40	511	49 (9.59%)	462 (90.41%)	1.43 (0.96-2.14)	2.84*	0.07
41-60	429	33 (7.69%)	396 (92.31%)	0.96 (0.62-1.47)	0.01*	0.84
>60	233	8 (3.43%)	225 (96.57%)	0.37 (0.18-0.76)	7.05*	0.005
Sex						
Female	383	13 (3.39%)	370 (96.61%)	0.33 (0.18-0.59)	14.23*	0.0001
Male	944	92 (9.75%)	852 (90.25%)			

Table 3 shows the modifiable patient factors associated with SSI. Among smokers, 10.76% had developed SSI, whereas among non-smokers, 6.12% developed the same. Therefore, the habit of smoking was found to have a highly statistically significant association with a p-value of 0.002.

**Table 3:** Modifiable patient factors associated with SSI the column total percentages are shown in brackets; *applying chi-square test, degree of freedom=1; ** applying Fisher exact test CI: confidence interval; SSI: surgical site infection

Risk factors	Total no. of patients (1327)	Patients with SSI (105)	Patients with no SSI (1222)	Odds ratio (95% CI)	Tests of association	P-value
Co-morbidities						
None	946	81 (8.56%)	865 (91.44%)	1.39 (0.87 -2.23)	1.61*	0.16
Diabetes	218	12 (5.5%)	206 (94.5%)	0.64 (0.34 -1.18)	1.7*	0.15
Hypertension	107	4 (3.74%)	103 (96.26%)	0.43 (0.16 -1.19)	2.19*	0.09
Diabetes and hypertension	56	8 (14.29%)	48 (85.71%)	2.01 (0.93 -4.39)	0.08**	0.08
Habit of smoking						
Yes	511	55 (10.76%)	456 (89.24%)	1.85 (1.24 -2.75)	8.64*	0.002
No	816	50 (6.13%)	766 (93.87%)			

Table 4 shows the preoperative and intraoperative factors associated with SSI. Around 6.9% of the patients with preoperative hospital stays of ≤3 days and 14.7% with >3 days of preoperative hospitalization had developed SSI. The length of preoperative hospital stay was found to be statistically significantly associated with the development of SSI with a p-value of 0.004. Since it was a tertiary care hospital, all patients who underwent orthopedic surgery received preoperative antimicrobial prophylaxis, hair removal was done with clippers, and skin preparation with iodine and alcohol was done; none were seen without these measures, hence no tests of statistical association to see the difference were done. Around 8.63% of SSI had ASA I, and 6.8% had ASA II. None of them with ASA 3 reported SSI; this difference between the association of the ASA score with the development of SSI was found to be statistically significant (p=0.04).

**Table 4:** Preoperative and intraoperative factors associated with SSI the column total percentages are shown in brackets; *applying chi-square test, degree of freedom=1; ** applying Fisher exact test ASA: American Society of Anesthesiologists score; CI: confidence interval; SSI: surgical site infection; NA: not applicable

Variable	Total no. of patients (1327)	Patients with SSI (105)	Patients without SSI (1222)	Odds ratio (CI)	P-value
Preoperative duration of hospitalization (in days)					
≤3	1158	80 (6.91%)	1078 (93.09%)	0.43 (0.26 -0.89)	0.004*
>3	169	25 (14.79%)	144 (85.21%)		
Antimicrobial prophylaxis					
Yes	1327	105 (7.91%)	1222 (92.09%)	-	NA
No	0	0	0		
Hair removal					
Yes	1327	105 (7.91%)	1222 (92.09%)	-	NA
No	0	0	0		
Skin preparation					
Iodine	0	0	0	-	NA
Iodine+ Isopropyl alcohol	1327	105 (7.91%)	1222 (92.09%)		
ASA					
I	950	82 (8.63%)	868 (91.37%)	1.45 (0.90-2.35)	0.12*
II	334	23 (6.89%)	311 (93.11%)	0.82 (0.51-1.32)	0.42*
III	43	0 (0%)	43 (100%)	-	0.04**
Duration of surgery (in minutes)					
60-180	347	22 (6.34%)	325 (93.66%)	0.73 (0.45-1.19)	0.21*
>180	980	83 (8.47%)	897 (91.53%)		
Cleanliness of the surgical site					
Clean	769	44 (5.72%)	725 (94.28%)	0.49 (0.32-0.74)	0.005*
Contaminated	558	61 (10.93%)	497 (89.07%)		
Number of people in the OT					
≤7	0	0	0	-	NA
>7	1327	105(7.91%)	1222 (92.09%)		

Out of 105 clinically diagnosed cases of SSI, 101 (96.19%) were culture positive, and four (3.80%) were culture negative. Among the sample population, 69.52% (73/105) developed SSI during the hospital stay, while 32.48% (32/105) reported the same after discharge. Among the patients who developed SSI, 91 (86.67%) had one organism, and 10 (9.52%) cases had two organisms in the microbial growth. Four (3.81%) had no microbial growth.

**Figure 3:**
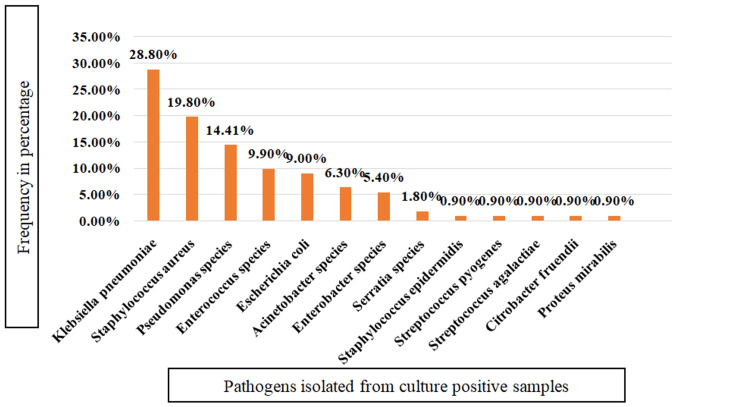
Frequency of various pathogens isolated from culture positive cases (total number of culture positive cases=101)

Figure 3 shows the frequency of various pathogens isolated from culture-positive cases. Out of 111 isolated organisms, 36 (25.7%) were gram-positive, and 75 (74.2%) were gram-negative. *Klebsiella pneumoniae* (32, 28.8%) was the most common isolate, followed by *Staphylococcus aureus* (22, 19.8%), *Pseudomonas *species (16, 14.41%), *Enterococcus* species (11, 9.9%), and *Escherichia coli* (10, 90%).

Table 5 shows the distribution of multidrug-resistant (MDR) organisms. Out of 75 gram-negative organisms, 54 (72%) were found to be MDR pathogens, and 31 (41%) were carbapenem-resistant gram-negative bacilli (CRGNB).

**Table 5:** Distribution of multidrug resistant bacterial pathogens in SSI Column total percentages are shown in brackets; #multi-drug resistant organism; *carbapenem resistant gram-negative bacilli SSI: surgical site infection

Bacterial pathogens	Frequencyin number (n),%	MDRO# in	Carbapenem resistant (CRGNB)*
Klebsiella pneumoniae	32 (47%)	30 (93%)	16 (53.3%)
Escherichia coli	10 (13%)	6 (60%)	4 (66%)
Enterobacter spp.	06 (8%)	2 (33.3%)	2 (100%)
Pseudomonas spp.	16 (21%)	12 (75%)	2 (16%)
Acinetobacter spp.	07 (9%0	4 (57%)	4 (100%)
Citrobacter spp	01 (1%0	1 (100%)	1(100%)
Serratia spp.	02 (3%)	2 (100%)	2 (100%)
Proteus spp.	01(1%0	0	0
Total = 75	75	54 /75 (72%)	31/75 (41%)

**Figure 4:**
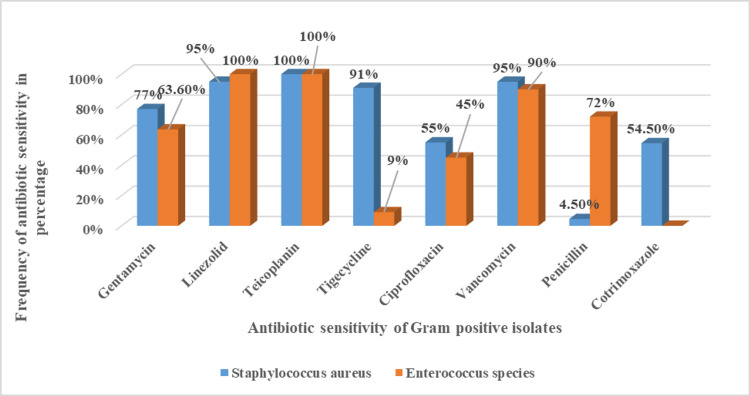
Antibiotic sensitivity pattern of gram-positive organisms (n=36)

Figure 4 shows the antibiotic sensitivity of gram-positive organisms. *Staphylococcus aureus* were sensitive to gentamicin (77%), vancomycin (90%), tigecycline (91%), linezolid (95%), and teicoplanin (100%), and 95.5% were resistant to penicillin, followed by 45% to ciprofloxacin and 45.5% to cotrimoxazole. *Enterococcus* species were sensitive to vancomycin (90%), teicoplanin (100%), and linezolid (100%) and were resistant to cotrimoxazole (100%), followed by tigecycline (91%), and were resistant to cotrimoxazole (100%) and tigecycline (91%) (Figure 4). Around 63.3% were methicillin-sensitive *Staphylococcus aureus *(MSSA), and 36.3% were methicillin-resistant *Staphylococcus aureus* (MRSA).

**Figure 5:**
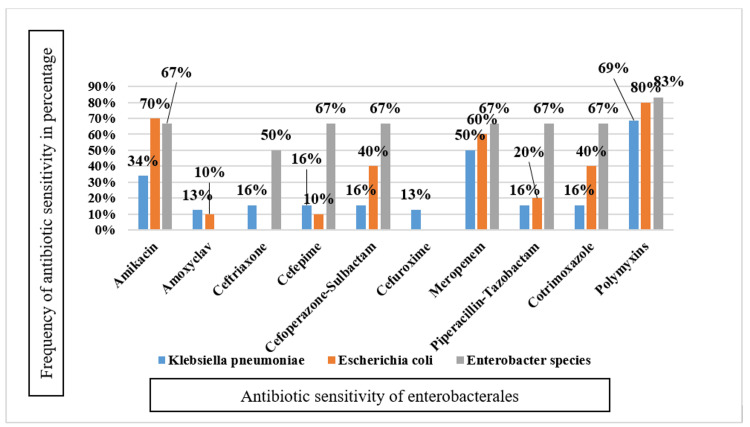
Sensitivity pattern of Enterobacterales among gram-negative isolates Out of 75 gram-negative isolates, 52 were *Enterobacterales*

*Klebsiella pneumoniae* were sensitive to polymyxins in 69% of cases and amikacin in 66% and resistant to amoxyclav (87%), cefuroxime (87%), ceftriaxone (84%), cefepime (84%), cefoperazone-sulbactam (84%), piperacillin-tazobactam (84%), and cotrimoxazole (84%) (Figure 5).

**Figure 6:**
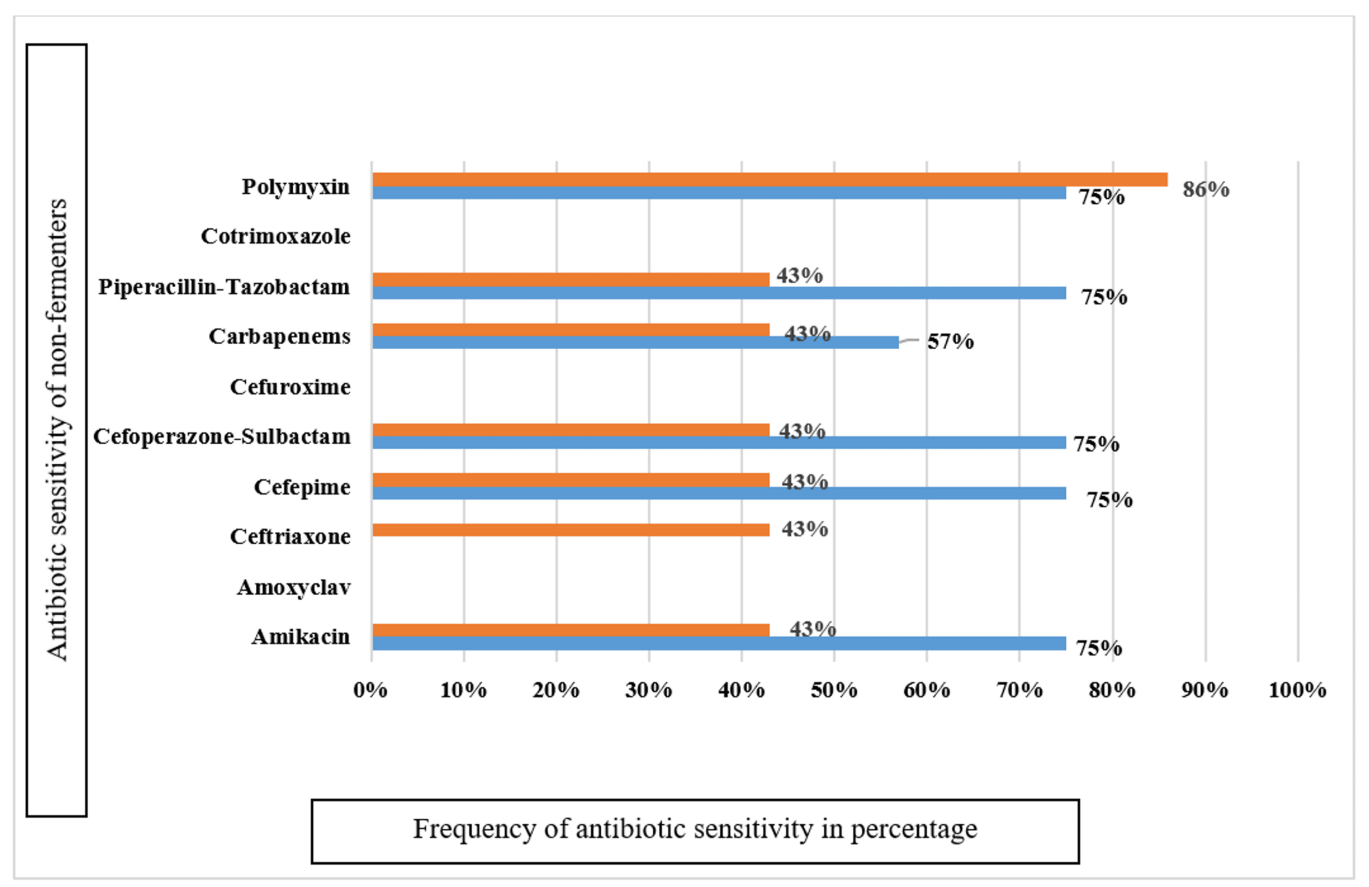
Sensitivity pattern of non-fermenters among gram-negative isolates Out of 75 gram-negative isolates, 23 were non-fermenters

Most of the *Pseudomonas* species were sensitive to polymyxins (75%) and amikacin (75%) and resistant to amoxicillin clavulanic acid (100%), cefuroxime (100%), and ceftriaxone (100%). *Acinetobacter* species were sensitive to polymyxins (86%) and 100% resistant to amoxicillin-clavulanic acid (Figure 6).

The mean duration of hospital stay in SSI patients was 10.73 ± 8.94 days, with a range of one day to 50 days, and that of non-SSI patients was 5.59 ± 1.86 days, with a range of one day to 11 days. This difference between the mean duration of hospital stay of those with SSI and those without SSI was also found to be statistically significant (p <0.0001). The median stay in the hospital for SSI patients was found to be nine days (IQR=5-13 days), while for non-SSI patients it was six days (IQR=4-7 days). Of all the patients followed up, death within one year of surgery was reported among 20 (18 deaths were reported among the patients having SSI and two deaths in the non-SSI group), although the exact reason related to that could not be assessed. The total number of readmissions was 208, with numbers in the SSI group being 74 of 105 (70.47%) and the non-SSI group 134 of 1222 (2.78%) (Table 6).

**Table 6:** Outcome of SSI * applying t-test; # applying Fischer’s exact test; ## applying chi-square test; **total number of deaths reported; the column total percentages are shown in brackets SD: standard deviation; SSI: surgical site infection

Parameter	SSI (n=105)	Non-SSI (n=1222)	P-value
Mean duration of hospital stay (days)	10.73 ± 8.94 SD	5.59 ± 1.86 SD	0.0001^*^
Death within 1 year after surgery (n=20**)	18 (90%)	2 (10%)	<0.0001^#^
Average cost during hospital stay			
Daily cost (in Indian rupees)	8821.78 ± 145.04 SD	4203.55 ± 193.15 SD	<0.001*
Total cost during hospital stay (in Indian Rupees)	170880.70 ± 51560.34 SD	24163.70 ± 9349.39 SD	<0.0001*
Frequency of re-admission			
One time (n=183)	53 (28.96%)	130(71.04%)	<0.0001^##^
Two times (n=19)	15 (78.95%)	4(21.05%)	<0.0001^#^
Three times (n=4)	4 (100%)	0(0%)	<0.0001^#^
Five times (n=1)	1 (100%)	0(0%)	0.12^#^
Six times (n=1)	1 (100%)	0 (0%)	0.12^#^

## Discussion

In our study, the overall SSI rate was 7.9%, and the number of SSIs was found to be highest at 34/359 (9.5%) in CRIF, followed by 65/766 (8.5%) in ORIF type of surgery, hip arthroplasty 3/69 (4.4%), and knee arthroplasty 3/133 (2.3%), which was similar to studies of Degbey et al. and Koyagura et al. (7.81% and 6.5% SSI rate) [7,8]. On the contrary, our findings were lower than those of studies conducted in various parts of India [9-13] and studies conducted in Addis Ababa, Ethiopia, with prevalence rates ranging from 54.3% to 82.4% [11,14]. The disparity of SSI rates could be explained by the incidence of bacterial etiology and infection prevention efforts in different hospital settings, study design, sample size, and population differences. Another possible explanation for the low rate of infection seen in our study is that the effect of antimicrobials used for surgical prophylaxis, the administration of antibiotics prior to obtaining culture samples, and antiseptics used for cleaning the wounds has been suggested to reduce the risk of infection and could be the major explanation for the absence of bacterial growth in samples taken from orthopedic surgical wounds with the existing clinical signs and symptoms of infection [11,14,15].

In this study, most of the patients were in the age group of 20-29 years (27.6%) with male preponderance (87%), whereas Suranigi et al. reported the most common age group to be 29-40 years (57.4%) with higher incidence in males (87.23%) [16]. In another study, Alelign et al. [17] found most of the SSIs to be in the age group 19-38 (44.9%) years with higher incidence in males (67.3%). The higher proportion of males in comparison to females (male-to-female ratio 7:1) in SSI cases can be explained by the fact that males, being more involved in outside activities and field occupations, are at higher risk of accidents with fractures. This predominance of males in operated patients has been previously illustrated in other studies [8,18,19]. Similar explanations can be applied to the prevalence of SSI in the lower age group rather than older patients [20].

Although maximum incidence of SSI (27.6%) was found in the 20-29 years age group, age >60 years was found to be statistically significant for the development of SSI in our study. It may be because of low immunity, increasing catabolism, comorbidities, and low wound healing rates in patients above 60 years of age [21-26]. Stephen Apanga et al. [22], Aikaterini et al. [23], Ibtesam et al. [24], Akinyoola et al. [25], and Khan et al. [26] also observed similar findings.

In our study, the maximum number of surgical procedures was ORIF (57.72%), and the highest SSI rate was found in CRIF (9.5%), followed by ORIF (8.5%), which is similar to the studies of Al-Mulhim et al. [27] and Kimmatkar N et al. [28], who reported maximum numbers of SSIs from CRIF, which were 54% and 22.27%, respectively, whereas Mathur et al. got the maximum number of SSI cases (58%) from ORIF surgical procedures [29]. Elifranji et al. [30] and Walaszek et al. [31] found the highest numbers of SSIs in hip arthroplasties, 14.5% and 35%, respectively. In our study, the majority of the surgeries were trauma surgeries (ORIF, CRIF), which is similar to the findings of Elifranji et al. [30] and Mathur [29]; the SSI rate is higher in trauma surgeries compared to arthroplasties, as preoperative soft tissue damage is a major risk factor for the development of SSI.

In the present study, the most prevalent comorbidities seen in SSI cases were diabetes mellitus (19.04%) followed by hypertension (11.42%), but no statistically significant association was found with the development of SSI. Our finding goes hand in hand with Lee et al. [20] and Wukich et al. [32], who reported diabetic patients among SSI cases to be 15% and 19%, respectively. Also, Le et al. [33] found diabetes and hypertension as significant comorbidities for SSI. Suranigi et al. [16] found 6.38% diabetic and 6.38% hypertensive patients among the SSI cases. Elifranji et al. [30] found 26.6% of SSI cases with hypertension and diabetes mellitus (24%). Significant association of diabetes mellitus was found with SSI in several studies by Kimmatkar et al. [28], Patel et al. [34], and Yang K et al. [35]. In diabetic patients, colonization and reproduction of certain bacteria are accelerated in high concentrations of glucose in tissue. So, diabetes is considered a modifiable variable in preventing postoperative SSI.

Around 53.3% of the SSI patients were smokers, which was a statistically highly significant factor for the development of SSI (p=0.0003). A similar finding was seen in other studies where 36.1% (Suranigi et al.) [16], 13% (Thakore RV et al.) [36], and 8% (Nota et al.) [37] were smokers in SSI cases. Durand et al. [38] found smoking to be an independent risk factor in the development of SSI, with 72% of the cases. Cigarette smoking has been reported to have an impact on wound healing through impairment of tissue oxygenation and local hypoxia via vasoconstriction [38-40].

Our study shows a higher incidence of SSI with a longer duration of surgery, >3 hours (79.04%), and no statistical significance was found with the duration of surgery and the development of SSI. A similar finding was reported by Koyagura et al. [8], where 60.9% of SSI cases had a duration of surgery >3 hours. Wukich et al. [32] and Le et al. [33] found an association between a longer duration of surgery (>132 mins) and SSI (p <0.05). In other studies, a duration of surgery longer than two hours resulted in 36% (Mathur et al.) [29] and 24.5% (Alelign et al.) [17] SSI cases, whereas, in contrast to our finding, Suranigi et al. [16] reported that 70% of cases occurred in patients having surgery of <3 hours.

In our study, 76% of the SSI cases had a preoperative hospital stay of >3 days and were found to be a statistically significant factor for the development of SSI (p=0.0005). In some studies, this association has also been mentioned as significant [41-45], whereas Suranigi et al. [16] found 29.78% of cases to have a preoperative hospital stay of >3 days and Boer et al. [46] reported 4.3% of cases having a preoperative hospital stay of >4 days. Prolonged preoperative stay increases the risk of colonization of the surgical site with bacteria and raises the risk of infection [47].

Patients with SSI in our study had ASA score I (78.09%) and ASA score II (21.9%). AL-Mulhim et al. [27] found SSI cases had ASA I (62%), ASA II (26.6%), and ASA III (11.4%). Lee et al. [20] found a higher incidence of SSI with ASA scores >III (61%). Thakore RV et al. [36] reported SSI in patients having ASA I (9%), ASA II (56%), and ASA III (31%). Mathur et al. [29] reported patients with ASA I (74.4%), ASA II (19%), and ASA III (6.3%). ASA score was not found to be statistically significant; however, clinical significance might be present. In our study, the majority of patients with infection had an ASA score of 1, but other studies have suggested that the higher the ASA score, the higher the risk of infection [48-50].

The risk factors that were statistically significant for the development of SSI were age >60 years (P=0.007), male gender (P=0.0002), smoking (P=0.0003), contaminated type of wound (P=0.0005), and length of preoperative hospital stay >3 days (P=0.0005). Koyagura et al. [8] found increased age >50 years and wound contamination to be significant, whereas Suranigi et al. [16] found preoperative hospital stay >3 days (P=0.02), and Durand et al. [38] reported smoking to be statistically significant factors for the development of SSI (P<0.001).

In the current study, 69.52% of the sample population developed SSI during their hospital stay, and around 30.47% developed the same after discharge from the hospital, whereas Walaszek et al. [41] found comparable numbers of cases of SSI diagnosed before and after discharge to be 52% and 48%, respectively. In contrast to our study, Mathur et al. [29] reported that 34% of patients were diagnosed with SSI during their hospital stay, and 66% of patients were diagnosed with SSI during the post-discharge period. Tuon et al. [51] suggests that most infections occur after hospital discharge and can be related to poor wound care by patients, but in our study, the higher number of patients were diagnosed during their hospital stay, which can be explained by various factors such as fecal contamination due to inadequate personnel hygiene or post-procedural contamination [47,52].

Culture positivity of samples in our study was 96.19%, whereas Al-Rashdi et al. [27] found 67.05% culture-positive samples. Suranigi et al. [16] reported 93.61% culture-positive samples from clinically diagnosed cases of SSI.

A total of 111 pathogens were isolated from all cases. *Klebsiella pneumoniae* (32, 28.8%) was the most common isolate, followed by *Staphylococcus aureus* (22, 19.8%), *Pseudomonas* species (16, 14.41%), *Enterococcus* species (11, 9.9%), *Escherichia coli* (10, 9%), and *Acinetobacter* *spp*. (7, 6.3%). This was close to the study by Al-Rashdi et al. [53], where the more common isolate was *Pseudomonas* species (25%), followed by *Enterobacterales* (23.61%), and the study of Rajkumari N et al. [54], where the predominant isolates were *Acinetobacter baumannii, Klebsiella pneumoniae*, and *Pseudomonas aeruginosa*.

The high incidence of gram-negative bacilli is common in developing countries, mainly *Enterobacterales*, followed by *Pseudomonas* species and *Acinetobacter baumannii *[55,56]. But Koyagura et al. [7] reported MRSA (48.4%) as the most common isolate, followed by Staphylococcus epidermidis (11.7%) and *Pseudomonas spp.* (10.9%). Mathur et al. [29] reported *Staphylococcus aureus* (36%) as the commonest organism isolated, followed by *Escherichia coli* (12%) and *Klebsiella pneumoniae* (10%). Tuon et al. [51] reported Staph aureus (34%) as the commonest isolate, followed by *Enterobacter* *spp*. (14.9%), *Pseudomonas aeruginosa* (11.6%), and *Serratia spp*. (8.2%). In another study, Kimmatkar et al. [28] reported the predominant organisms as *Staphylococcus aureus *(54.54%), followed by *Escherichia coli *(18.18%) and *Klebsiella* species (8.18%). In our study, 74% were gram-negative organisms and 25.7% were gram-positive organisms, which is similar to observations of Al-Rashdi et al. [53] and Toledo et al. [55], and in contrast to the study of Elifranji et al. [30], where 70% of isolates were gram-positive organisms. The high prevalence rate of gram-negative bacterial isolates could indicate fecal contamination as a result of inadequate personnel hygiene or post-procedural contamination, as well as the high prevalence of gram-negative bacteria as normal flora in the hospital setting [47].

Around 9.52% of the samples in our study showed polymicrobial growth. In other studies, polymicrobial growth was seen in 9.09% (Kimmatkar et al.) [28], 20.8% (Vickers et al.) [56], and 29.78% (Pull ter Gunne et al.) [57] of the samples from SSI cases.

In our study, among the gram-negative organisms, 54 (72%) were MDROs, and 41% were carbapenem-resistant gram-negative bacilli. Al-Rashdi [53] and Vickers et al. [56] reported 37.51% and 38.8%, respectively, of MDROs among gram-negative bacteria. Also, Alelign et al. [17] found multi-drug-resistant status in 39.5% of gram-negative organisms isolated. In our study, among the *Staphylococcus aureus* isolates, 36% were methicillin-resistant *Staphylococcus aureus*. Koyaguru et al. [8] reported MRSA in 48.4% of the cases, and Pull ter Gunne et al. [57] reported a 62% incidence of MRSA isolates. Alelign et al. [17] found 57.9% MRSA among the *Staphylococcus aureus* isolates. This difference and increased prevalence of MDROs can be explained by different bacterial strains, geographic variations, patients’ awareness towards the use of antimicrobials, the difference in hospital infection control measures, the easy availability of drugs without prescriptions, and the indiscriminate use of common antibiotics, leading to rapid and extensive spread of antimicrobial resistance.

*Klebsiella pneumoniae* were mostly resistant to amoxiclav (87%) and cefuroxime (87%), followed by ceftriaxone (84%). They were sensitive to polymyxins (69%) and amikacin (66%). In another study done by Alelign et al. [17], they were 69.2% resistant to ampicillin, cefepime, cefoxitin, ceftriaxone, cefuroxime, and ciprofloxacin. Tuon et al. [51] observed 100% resistance of *Klebsiella pneumoniae* to ampicillin-sulbactam, cefepime, and cotrimoxazole. Furthermore, Elifranji et al. [30] observed high resistance of *Klebsiella pneumoniae* against co-trimoxazole (80%), cefazolin (100%), and ampicillin (100%) and responsiveness to meropenem (100%) and polymyxin (100%).

Most of the *Pseudomonas* species were resistant to amoxicillin clavulanic acid (100%), cefuroxime (100%), and ceftriaxone (100%) and sensitive to polymyxins (75%) and amikacin (75%) in our study, whereas Alelign et al. [17] observed resistance of 70% of isolates to cefepime, ceftazidime, and ciprofloxacin and susceptibility to piperacillin (60%) and meropenem (60%). In another study, Tuon et al. [51] reported 100% susceptibility of *Pseudomonas* species to amikacin, ciprofloxacin, meropenem, polymyxins, and cefepime (81%) and ceftazidime (93%).

Among gram-positive isolates, *Staphylococcus aureus* were 95.5% resistant to penicillin, followed by 45% to ciprofloxacin and 45.5% to cotrimoxazole. They were sensitive to gentamicin 77%, vancomycin 90%, tigecycline 91%, linezolid 95%, and teicoplanin 100%, whereas Alelign et al. [16] showed 78.9%, 63.2%, and 57.9% resistance to penicillin, cotrimoxazole, and erythromycin, respectively. In a similar study by Mathur et al. [29], *Staphylococcus aureus* were 100% resistant to penicillin, followed by ciprofloxacin (94.1%), levofloxacin (93.7%), and sensitive to gentamycin (70.6%), cefoxitin (71.4%), and clindamycin (64.7%), daptomycin (100%), and linezolid (100%), whereas Tuon et al. [51] reported sensitivity to ampicillin-sulbactam (88%), cefazolin (88%), cefepime (88%), ciprofloxacin (88%), meropenem (88%), cotrimoxazole (96%), and vancomycin (100%).

Mean duration of hospital stay in SSI patients was 10.73 ± 8.94 with a range of one day to 50 days, which was (P <0.0001), which was similar to the finding of Mathur et al. [29] and Whitehouse et al. [47].

Furthermore, Lee et al. [20] also reported the median length of hospital stay of SSI patients to be 13 days with a mean of nine days attributable to the infection.

The average daily cost of treatment in hospitals for SSI patients is Rs. 8821.78 ± 145.04, whereas for non-SSI individuals it is Rs. 4203.55 ± 193.15. Our study shows that cost attributable to SSI is almost double the cost of treatment of a patient without SSI and is statistically significant (P <0.001). Our finding is similar to the finding of Thakore RV et al. [36], and Mathur et al. [29] also found cost attributable to SSI to be a significant outcome (P <0.0001). Similar results regarding the cost of treatment were observed in other studies by Whitehouse et al. [47], Sculco et al. [54], and Hebert et al. [58].

SSI patients were readmitted more times compared to the non-SSI patients. Similar findings were reported by Mathur et al. [29], Pawlowska et al. [59], and Whitehouse et al. [47].

Limitations of the study

Sample size is small, as it is a single-centered study, but a larger sample size might be possible with a multicenter approach. Death attributed to SSI within one year of surgery may be due to other causes also. Other variables not controlled for are as follows: type of implants used and miscellaneous host-dependent variables, postoperative patient compliance, wound surveillance, and home care.

## Conclusions

Surgical site infections are frequently encountered complications following surgical procedures. Even with the most advanced surgical equipment and operation theatres, careful sterilization procedures, and preoperative antibiotic prophylaxis, SSIs remain a significant source of HAIs among patients undergoing orthopedic surgeries. SSI continues to be a significant financial and physical burden for the patient. Appropriate preoperative, intraoperative, and postoperative patient care, suitable infection control methods, and a sensible antibiotic policy can help to lower the SSI rate. As the empirical treatment of HAIs provokes drug resistance, treatment should be based on the culture and sensitivity report, and continuous surveillance for resistant bacteria should be conducted to prevent the spread of MDR bacterial infections. The higher rates of SSI in the orthopedic unit may be due to inadequate infection control. So, active surveillance of SSI will help in early management and prevention.

## References

[1] World Health Organization (2018). Global Guidelines for the Prevention of Surgical Site Infection. Global Guidelines for the Prevention of Surgical Site Infection.

[2] Mathur P (2018). Prevention of healthcare-associated infections in low- and middle-income Countries: the 'bundle approach'. Indian J Med Microbiol.

[3] Durlach R, McIlvenny G, Newcombe RG (2012). Prevalence survey of healthcare-associated infections in Argentina; comparison with England, Wales, Northern Ireland and South Africa. J Hosp Infect.

[4] Raffaldi I, Scolfaro C, Pinon M (2011). Surveillance study of healthcare-associated infections in a pediatric neurosurgery unit in Italy. Pediatr Neurosurg.

[5] Lilani SP, Jangale N, Chowdhary A, Daver GB (2005). Surgical site infection in clean and clean-contaminated cases. Indian J Med Microbiol.

[6] Berríos-Torres SI, Umscheid CA, Bratzler DW (2017). Centers for Disease Control and Prevention Guideline for the Prevention of Surgical Site Infection, 2017. JAMA.

[7] Dégbey C, Kpozehouen A, Coulibaly D, Chigblo P, Avakoudjo J, Ouendo EM, Hans-Moevi A (2021). Prevalence and factors associated with surgical site infections in the university clinics of traumatology and urology of the National University Hospital Centre Hubert KoutoukouMaga in Cotonou. Front Public Health.

[8] Koyagura B, Koramutla HK, Ravindran B, Kandati J Surgical site infections in orthopaedic surgeries: incidence and risk factors at tertiary care hospital of South India. Int J Res Orthop2018.

[9] Sarangi SK, Padhi S (2019). Bacteriological profile of post-operative orthopedic implant infections and their antibiotic sensitivity pattern in a tertiary care hospital of southern Odisha. J Dr NTR Univ Health Sci.

[10] Fernandes A, Dias M (2013). The microbiological profiles of infected prosthetic implants with an emphasis on the organisms which form biofilms. J Clin Diagn Res.

[11] Mezemir R, Seid A, Gishu T, Demas T, Gize A (2020). Prevalence and root causes of surgical site infections at an academic trauma and burn center in Ethiopia: a cross-sectional study. Patient Saf Surg.

[12] Shabnum M, Vasundhara DP, Sreenivasulu RP (2017). Microbial profile and antibiotic susceptibility pattern of orthopedic infections in a tertiary care hospital: a study from South India. Int J Med Sci Public Health.

[13] Benazir S, Kakru DK, Khurshid S (2018). Identification, antibiotic susceptibility patterns and biofilm detection of isolates in orthopaedic implant infections. J adv med.

[14] Asres G, Legese M, Woldearegay G (2017). Prevalence of multidrug resistant Bacteria in postoperative wound infections at TikurAnbessa Specialized Hospital, Addis Ababa, Ethiopia. Arch Med.

[15] Lakshminarayana S, Chavan S, Prakash R, Sangeetha S (2013). Bacteriological profile of orthopedic patients in a tertiary care hospital, Bengaluru. Int J Sci Res.

[16] Suranigi SM, Ramya SR, Sheela Devi C, Kanungo R, Najimudeen S (2021). Risk factors, bacteriological profile and outcome of surgical site infections following orthopaedic surgery. Iran J Microbiol.

[17] Alelign D, Tena T, Tadesse D (2022). Bacteriological profiles, antimicrobial susceptibility patterns, and associated factors in patients undergoing orthopedic surgery with suspicion of surgical site infection at Arba Minch General Hospital in Southern Ethiopia. Infect Drug Resist.

[18] Kumar S, Sengupta M, Hada V, Sarkar S, Bhatta R, Sengupta M (2017). Early post-operative wound infection in patients undergoing orthopaedic surgery with implant. Int J Sci Study.

[19] Mardanpour K, Rahbar M, Mardanpour S, Mardanpour N (2017). Surgical site infections in orthopedic surgery: incidence and risk factors at an Iranian teaching hospital. Clin Trials OrthopDisord.

[20] Lee J, Singletary R, Schmader K, Anderson DJ, Bolognesi M, Kaye KS (2006). Surgical site infection in the elderly following orthopaedic surgery. Risk factors and outcomes. J Bone Joint Surg Am.

[21] Bandaru NR, A RR, K VP, DVSS RM (2012). A prospective study on the postoperative wound infections. Journal of Clinical and Diagnostic Research.

[22] Apanga S, Adda J, Issahaku M, Amofa J, Mawufemor KR (2013). Epidemiology of wound infection in a surgical ward at a tertiary care hospital in Northern Ghana. Int J Med Health Sci.

[23] Masgala A, Chronopoulos E, Nikolopoulos G (2012). Risk factors affecting the incidence of infection after orthopaedic surgery: the role of chemoprophylaxis. Cent Eur J Public Health.

[24] Afifi IK, Baghagho EA (2010). Three months study of orthopaedic surgical site infections in an Egyptian University hospital. International Journal of Infection Control.

[25] Akinyoola AL, Adegbehingbe OO, Ogundele OJ (2008). Factors influencing the outcome of elective paediatric orthopaedic operations in Ile-Ife, Nigeria. Tanzan J Health Res.

[26] Khan MS, ur Rehman S, Ali MA, Sultan B, Sultan S (2008). Infection in orthopedic implant surgery, its risk factors and outcome. J Ayub Med Coll Abbottabad.

[27] Al-Mulhim FA, Baragbah MA, Sadat-Ali M, Alomran AS, Azam MQ (2014). Prevalence of surgical site infection in orthopedic surgery: a 5-year analysis. Int Surg.

[28] Kimmatkar N, Hemnani JT (2017). Incidence of surgical site infections in IPD orthopedics patients undergoing implant surgery. Int Arch BioMed Clin Res.

[29] Mathur P, Mittal S, Trikha V (2022). Surveillance for surgical site infections in orthopedic trauma surgeries at an Indian hospital. Indian J Med Microbiol.

[30] Elifranji ZO, Haddad B, Salameh A (2022). Microbiological profile and drug resistance analysis of postoperative infections following orthopedic surgery: a 5-year retrospective review. Adv Orthop.

[31] Wałaszek MZ, Słowik R, Domański A, Wałaszek MJ, Różańska A, Kołpa M (2022). Five-year analysis of surgical site infections in three orthopaedics and trauma wards under HAI-net from the south of Poland in 2014-2018 considering the standardized infection ratio. Medicina (Kaunas).

[32] Wukich DK, Lowery NJ, McMillen RL, Frykberg RG (2010). Postoperative infection rates in foot and ankle surgery: a comparison of patients with and without diabetes mellitus. J Bone Joint Surg Am.

[33] Le J, Dong Z, Liang J, Zhang K, Li Y, Cheng M, Zhao Z (2020). Surgical site infection following traumatic orthopaedic surgeries in geriatric patients: Incidence and prognostic risk factors. Int Wound J.

[34] Patel SM, Patel MH, Patel SD, Soni ST, Kinariwala DM, Vegad MM (2012). Surgical site infections: Incidence and risk factors in a tertiary care hospital, Western India. National Journal of Community Medicine.

[35] Yang K, Yeo SJ, Lee BP, Lo NN (2001). Total knee arthroplasty in diabetic patients: a study of 109 consecutive cases. J Arthroplasty.

[36] Thakore RV, Greenberg SE, Shi H (2015). Surgical site infection in orthopedic trauma: a case-control study evaluating risk factors and cost. J Clin Orthop Trauma.

[37] Nota SP, Braun Y, Ring D, Schwab JH (2015). Incidence of surgical site infection after spine surgery: what is the impact of the definition of infection?. Clin Orthop Relat Res.

[38] Durand F, Berthelot P, Cazorla C, Farizon F, Lucht F (2013). Smoking is a risk factor of organ/space surgical site infection in orthopaedic surgery with implant materials. Int Orthop.

[39] Nolan MB, Martin DP, Thompson R, Schroeder DR, Hanson AC, Warner DO (2017). Association between smoking status, preoperative exhaled carbon monoxide levels, and postoperative surgical site infection in patients undergoing elective surgery. JAMA.

[40] Sørensen LT, Jørgensen S, Petersen LJ, Hemmingsen U, Bülow J, Loft S, Gottrup F (2009). Acute effects of nicotine and smoking on blood flow, tissue oxygen, and aerobe metabolism of the skin and subcutis. J Surg Res.

[41] Haidara DB (2021). Study of factors associated with surgical wound infections at the Ouidah Zone Hospital, Benin (Article in French). Mali Sante Publique [Internet].

[42] Hodonou MA, Hounkponou F, Allodé SA (2016). Bacteriological aspects of surgical site infections at the Borgou Departmental Hospital Center in Parakou (Benin) (Article in Frech). EurSci J.

[43] Ouendo EM, Saizonou J, Degbey C, Glele K, Glele Y, Makoutode M (2015). Management of infectious risk associated with care and services at the Hubert Koutoukou Maga National Hospital and University Center in Cotonou (Benin) (Article in French). Int J Biol Chem Sci.

[44] Ouédraogo AS, Somé DA, Dakouré PW, Sanon BG, Birba E, Poda GE, Kambou T (2011). Bacterial profile of surgical site infections at Souro Sanou National Hospital Center in Bobo Dioulasso, Burkina Faso (Article in Frech). Med Trop (Mars).

[45] McGarry SA, Engemann JJ, Schmader K, Sexton DJ, Kaye KS (2004). Surgical-site infection due to Staphylococcus aureus among elderly patients: mortality, duration of hospitalization, and cost. Infect Control Hosp Epidemiol.

[46] de Boer AS, Mintjes-de Groot AJ, Severijnen AJ, van den Berg JM, van Pelt W (1999). Risk assessment for surgical-site infections in orthopedic patients. Infect Control Hosp Epidemiol.

[47] Whitehouse JD, Friedman ND, Kirkland KB, Richardson WJ, Sexton DJ (2002). The impact of surgical-site infections following orthopedic surgery at a community hospital and a university hospital: adverse quality of life, excess length of stay, and extra cost. Infect Control Hosp Epidemiol.

[48] Bachoura A, Guitton TG, Smith RM, Vrahas MS, Zurakowski D, Ring D (2011). Infirmity and injury complexity are risk factors for surgical-site infection after operative fracture care. Clin Orthop Relat Res.

[49] Maksimović J, Marković-Denić L, Bumbasirević M, Marinković J, Vlajinac H (2008). Surgical site infections in orthopedic patients: prospective cohort study. Croat Med J.

[50] Culver DH, Horan TC, Gaynes RP (1991). Surgical wound infection rates by woundclass, operative procedure, and patient risk index. Am J Med.

[51] Tuon FF, Cieslinski J, Ono AF (2019). Microbiological profile and susceptibility pattern of surgical site infections related to orthopaedic trauma. Int Orthop.

[52] Mama M, Abdissa A, Sewunet T (2014). Antimicrobial susceptibility pattern of bacterial isolates from wound infection and their sensitivity to alternative topical agents at Jimma University Specialized Hospital, South-West Ethiopia. Ann Clin Microbiol Antimicrob.

[53] Al-Rashdi A, Al Wahaibi A, Al-Yaqoubi MM (2020). Surgical site infections after orthopedic procedures in a tertiary hospital in Oman: incidence, characteristics and risk factors. Gazette of Medical Sciences.

[54] Sculco TP (1993). The economic impact of infected total joint arthroplasty. Instr Course Lect.

[55] Toledo PV, Arend LN, Pilonetto M, Costa Oliveira JC, Luhm KR (2012). Surveillance programme for multidrug-resistant bacteria in healthcare-associated infections: an urban perspective in South Brazil. J Hosp Infect.

[56] Vickers ML, Ballard EL, Harris PN (2020). Bacterial profile, multi-drug resistance and seasonality following lower limb orthopaedic surgery in tropical and subtropical Australian hospitals: an epidemiological cohort study. Int J Environ Res Public Health.

[57] Pull ter Gunne AF, Cohen DB (2009). Incidence, prevalence, and analysis of risk factors for surgical site infection following adult spinal surgery. Spine (Phila Pa 1976).

[58] Hebert CK, Williams RE, Levy RS, Barrack RL (1996). Cost of treating an infected total knee replacement. Clin Orthop Relat Res.

[59] Pawłowska I, Ziółkowski G, Wójkowska-Mach J, Bielecki T (2019). Can surgical site infections be controlled through microbiological surveillance? A three-year laboratory-based surveillance at an orthopaedic unit, retrospective observatory study. Int Orthop.

